# Supplementary material for: Enhanced susceptibility to predation in corals of compromised condition
Source: PeerJ. 2015 Sep 10;3:e1239. doi: 10.7717/peerj.1239 (PMC4699786; doi:10.7717/peerj.1239)
Supplement: Table S1 — (A) The number of successful (i.e., a choice was made) and unsuccessful trials (i.e., no choice was made) conducted during daytime and nighttime for snails sourced from Acropora palmata (Ap) and A. cervicornis (Ac). (B) The percent of successful trials conducted during daytime and nighttime. [file peerj-03-1239-s001.docx]

|  | A | | | | | | | | B | | | | | |
| --- | --- | --- | --- | --- | --- | --- | --- | --- | --- | --- | --- | --- | --- | --- |
|  | *Acropora palmata* Sourced Snails | | | | *Acropora cervicornis* Sourced Snails | | | | % of Successful Trials | | | | | |
|  | Day | | Night | | Day | | Night | | Ap | | | Ac | | |
| Trial Type | choice | no choice | choice | no choice | choice | no choice | choice | no choice | Day | Night | p | Day | Night | p |
| H v D | 3 | 13 | 20 | 4 | 14 | 4 | 15 | 7 | 19% | 83% | < 0.0001 | 78% | 68% | ns |
| H v M | 2 | 8 | 24 | 7 | 0 | 0 | 32 | 7 | 20% | 77% | < 0.0001 | - | 82% | - |
| H v S | 3 | 25 | 14 | 19 | 12 | 6 | 16 | 4 | 11% | 42% | < 0.0001 | 67% | 80% | ns |
| H v P | 0 | 0 | 2 | 0 | 1 | 0 | 0 | 0 | - | 100% | - | 100% | - | - |
| All Trials | 8 | 46 | 60 | 30 | 27 | 10 | 63 | 18 | 15% | 67% | < 0.0001 | 73% | 78% | ns |
