# Supplementary figures and images for: Enhanced susceptibility to predation in corals of compromised condition
Source: PeerJ. 2015 Sep 10;3:e1239. doi: 10.7717/peerj.1239 (PMC4699786; doi:10.7717/peerj.1239)

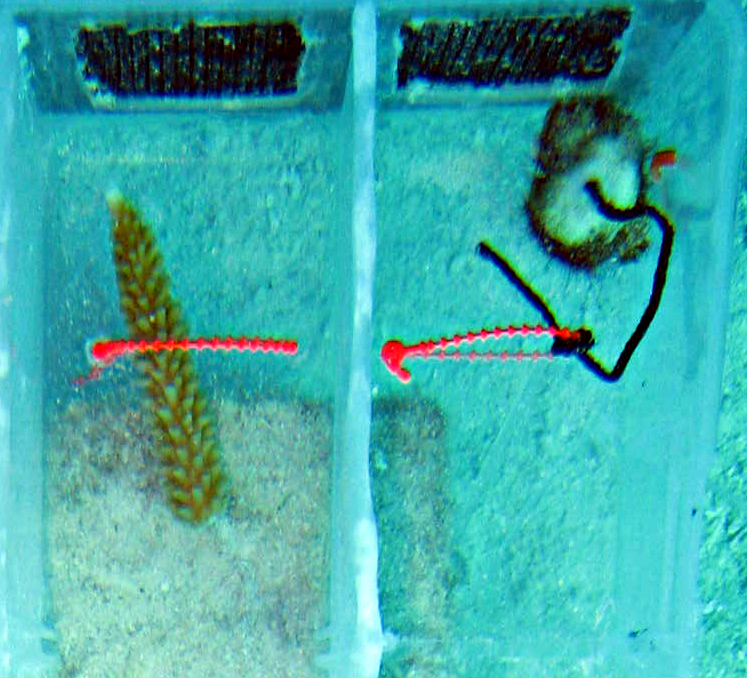

Supplement: Figure S1 — Photo example of a trial with a healthy coral fragment versus a solitary snail (H v S). [file peerj-03-1239-s002.jpg]
